# A qualitative evidence synthesis on the management of male obesity

**DOI:** 10.1136/bmjopen-2015-008372

**Published:** 2015-10-12

**Authors:** Daryll Archibald, Flora Douglas, Pat Hoddinott, Edwin van Teijlingen, Fiona Stewart, Clare Robertson, Dwayne Boyers, Alison Avenell

**Affiliations:** 1 Scottish Collaboration for Public Health Research & Policy (SCPHRP), University of Edinburgh, Edinburgh, Lothian, UK; 2 Rowett Institute of Health and Nutrition, University of Aberdeen, Medical School, Aberdeen, UK; 3 NMAHP Research Unit, Unit 13 Scion House, University of Stirling, Stirling University Innovation Park, Stirling, UK; 4 Maternal & Perinatal Health Faculty of Health & Social Sciences, Centre for Midwifery, Christchurch Road Bournemouth University, Bournemouth, UK; 5 Cochrane Incontinence Review Group, University of Aberdeen, Aberdeen, UK; 6 Health Services Research Unit (HSRU), University of Aberdeen, Aberdeen, UK; 7 Health Economics Research Unit (HERU) and Health Services Research Unit (HSRU), University of Aberdeen, Aberdeen, UK

**Keywords:** Men, Weight management, Systematic Reviews, Behavioural Change

## Abstract

**Objectives:**

To investigate what weight management interventions work for men, with which men, and under what circumstances.

**Design:**

Realist synthesis of qualitative studies.

**Data sources:**

Sensitive searches of 11 electronic databases from 1990 to 2012 supplemented by grey literature searches.

**Study selection:**

Studies published between 1990 and 2012 reporting qualitative research with obese men, or obese men in contrast to obese women and lifestyle or drug weight management were included. The studies included men aged 16 years or over, with no upper age limit, with a mean or median body mass index of 30 kg/m^2^ in all settings.

**Results:**

22 studies were identified, including 5 qualitative studies linked to randomised controlled trials of weight maintenance interventions and 8 qualitative studies linked to non-randomised intervention studies, and 9 relevant UK-based qualitative studies not linked to any intervention. Health concerns and the perception that certain programmes had ‘worked’ for other men were the key factors that motivated men to engage with weight management programmes. Barriers to engagement and adherence with programmes included: men not problematising their weight until labelled ‘obese’; a lack of support for new food choices by friends and family, and reluctance to undertake extreme dieting. Retaining some autonomy over what is eaten; flexibility about treats and alcohol, and a focus on physical activity were attractive features of programmes. Group interventions, humour and social support facilitated attendance and adherence. Men were motivated to attend programmes in settings that were convenient, non-threatening and congruent with their masculine identities, but men were seldom involved in programme design.

**Conclusions:**

Men's perspectives and preferences within the wider context of family, work and pleasure should be sought when designing weight management services. Qualitative research is needed with men to inform all aspects of intervention design, including the setting, optimal recruitment processes and strategies to minimise attrition.

**Strengths and limitations of this study**

- To the best of our knowledge, this is the first synthesis of qualitative studies investigating men's perceptions and experiences of weight management services.
- This qualitative evidence synthesis may help with the formation of policies to increase men's uptake and continuation with weight management services.
- Exhaustive searches were undertaken with the aim of identifying all relevant published and grey literature.
- The evidence base is limited for black or ethnic minority men; men who are unemployed or on low incomes; men living in rural and/or remote areas; and men who are gay, bisexual or transgender.

## Background

More men than women are overweight or obese in the UK, and this difference is projected to continue. In the Health Survey for England 2013,^1^ 41% of men were overweight compared with a figure of 33% in women, whereas 26% of men were obese compared with a figure of 24% in women. As the prevalence of obesity continues to increase, it is likely that people who are overweight will become obese in the future; indeed 47% of men and 36% of women by 2025 are predicted to become obese in England by 2025.^2^

Obesity is a risk factor for a very wide range of diseases, impacting on health and quality of life. Men with BMI (body mass index) ≥30 kg/m^2^ and waist circumference ≥102 cm have an increased risk of at least one symptom of impaired physical, psychological or sexual function, and these symptoms are also more likely in men with raised waist circumference but lower BMI.^3^

However, men are under-represented in weight loss programmes (WLPs). A recent systematic review^4^ found that only 27% of participants in randomised trials were men. In the UK, only 23% of participants in the counterweight WLP, involving 65 general practices, were men.^5^ Men comprise only around 10% of participants in commercial WLPs.^6^

Different ideas exist about why men are under-represented in WLPs. For example, it seems that men may be more reluctant to change their lifestyle than women, and be more cynical about government health messages.^7^ Media and other cultural influences encourage men to maintain a larger, muscular, masculine body size.^8^ In addition, men appear less interested in gaining an ideal body weight (by a medical definition) and more interested in physical activity and body shape.^9^ There are also differences in the way that men and women view physical activity as a means of becoming stronger, fitter and healthier.^10^

WLPs and facilities, including commercial weight loss organisations, can be seen as feminised spaces,^11^ while men prefer programmes in masculine spaces.^12^ Fear and embarrassment may particularly hinder men taking part in WLPs.^13^ Men are under-represented in such programmes, suggesting that methods to engage men in services, and the services themselves, are currently not appealing to men.

In order to develop a better understanding about what might improve men's uptake of weight management programmes, we undertook a realist integrated qualitative and quantitative evidence synthesis to investigate what weight management interventions work for men, with which men, and under what circumstances.^14^ This was underpinned by a socioecological theory of behaviour change.^15^ From a realist perspective, it is important to conceptualise any intervention intended to improve health by considering the:

1. Context that an intervention/programme will be situated within, so that factors that might inhibit or enhance its effectiveness, engagement and adherence can be identified;
2. Mechanisms of the intervention/programme and how the intended programme beneficiaries will interact and react to the intervention processes and mechanisms; and
3. Outcomes, both positive and negative, that may arise from an individual's engagement with the proposed intervention.

This qualitative evidence synthesis was conducted as part of the wider systematic (Review Of MEn and Obesity (ROMEO) project).^16^ This paper focuses on the main themes that emerged from the qualitative synthesis of included studies. These themes shed light on factors that were: (1) important or instrumental in encouraging men to engage in WLPs; (2) evident in men's perspectives and experiences of weight management programmes (once engaged) and (3) contained in their perspectives of the impacts and consequences of their engagement with such a programme.

## Methods

Comprehensive literature searches^17^ were undertaken to identify studies reporting qualitative research with obese men, or obese men in contrast to obese women, published since 1990 in any language. The following bibliographic databases were searched: Ovid MEDLINE (1948–2012 week 31) and MEDLINE-in-Process and other non-indexed citations (1948–30 July 2012), Ovid EMBASE (1980–2012 week 31), EBSCO CINAHL (1981–July 2012), EBSCO PsycINFO (1800–July 2012), Proquest Applied Social Sciences Index and Abstracts (1987–July 2012), Proquest Education Resources Information Center (1966–July 2012), OCLC Anthropology Plus (1957–July 2012), Ovid British Nursing Index (1994–July 2012), Social Sciences Citation Index (1970–July 2012), and Conference Proceedings Citation Index—Social Science and Humanities (1990–July 2012).

In addition, we included qualitative data from both health professionals and commercial organisations involved in managing obesity. Reference lists of all included studies were read to identify any additional potentially relevant reports. We also searched the internet for online WLPs specifically targeted at men and the Picker Institute and Joanna Briggs Institute websites for grey literature.

The ROMEO project^16^ identified studies reporting results of randomised controlled trials (RCTs) of weight management interventions recruiting men only. Where contact details were available, all authors of men-only RCTs were contacted to identify any qualitative or other relevant published or unpublished reports.

### Inclusion and exclusion criteria

Eligible studies included men who were 16 years or over, with no upper age limit, who were reported to have a mean or median BMI of 30 kg/m^2^ and reported primary qualitative research data. Studies conducted in developed countries were included if they contributed relevance to the UK context and all settings for lifestyle and drug interventions were considered.

### Identification of studies

Two researchers (DA and FD) independently screened abstracts for inclusion and read the full-text reports to select those for inclusion. In cases where there were uncertainties over the inclusion of a study, the wider ROMEO team met to discuss and decide. DA and FD then grouped the final included studies into three categories:

- Qualitative and mixed-method studies linked to eligible RCTs, including any qualitative data reported as part of papers reporting quantitative outcomes;
- Qualitative and mixed-method studies linked to ineligible RCTs and identified non-randomised intervention studies including any reported qualitative data reported;
- UK-based qualitative studies not linked to any specific interventions that contain men-only samples.

### Describing and appraising studies

A data extraction form was used to extract details of study design, methods, participants, interventions, findings, data pertaining to area and setting, and quality assessment. The data extraction form was developed around 10 detailed research questions. These emerged from (1) the systematic review of men-only RCTs conducted as part of the wider ROMEO project and (2) the expertise, knowledge and previous research of the research team along with our advisory group of representatives from the Men's Health Forums covering England and Wales, Scotland and All-Ireland:

1. How are men initially motivated to lose weight?
2. How are men attracted to taking part in the trial/intervention?
3. Are men consulted in the design of the intervention?
4. If it is found that interventions for men should be different from those for women, how should they be different and why?
5. Are group-based interventions for men found to be more effective for weight loss than those delivered to individual men?
6. Are certain features of diets found to be more attractive for obese men?
7. Are certain features of physical activity stated to be more attractive for obese men? How and why are these features more attractive?
8. What efforts are made to help men continue with the programme?
9. Do men state who they believe to be the best person/persons to deliver the intervention?
10. Are programmes deliberately involving partners/families more effective?

Generating inductive research questions in this way is an inherent property of qualitative research, and particularly of a grounded theory approach in which data collection and analysis proceed iteratively to confirm or refute an emerging theory. These questions were incorporated into the data extraction form to code the data to identify a priori themes. In addition, the data extraction form also contained sections where the reviewer could add detail on any emergent themes in the included studies that did not correspond to the a priori themes.

### Quality assessment

Our quality assessment tool combined the following critical appraisal checklists: Critical Appraisal Skills Programme,^18^ the Consolidated Criteria for Reporting Qualitative Research^19^ and the Joanna Briggs Institute Qualitative Assessment and Review Instrument.^20^ We included criteria from these that were key in terms of: (1) methodological rigour; and (2) importance for our a priori research questions, which were specifically concerned with informing policy and practice. We did not exclude studies on the basis of quality. This decision was influenced by recent arguments within the qualitative evidence synthesis methods literature. For example, recent studies^21^ ^22^ have argued that excluding studies because of methodological flaws or incomplete reporting may result in the loss of valuable new insights, whereas studies that are methodologically sound may suffer from poor interpretation of data, leading to an insufficient insight into the phenomenon under study. A full description of our approach to quality assessing included studies is available in our full HTA (Health Technology Assessment) report.^16^

### Analysis

**Figure 1:**
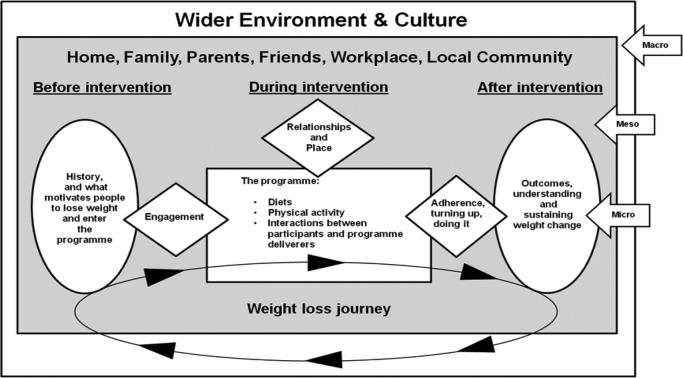
Review Of MEn and Obesity (ROMEO) evidence synthesis logic model.

We implemented a three-stage analysis cycle:

1. Two researchers (DA and FD) extracted data from the included studies linked to interventions and categorised the findings of these studies according to whether they corresponded to a priori themes or emergent themes unconnected to the a priori themes.
2. An initial descriptive thematic index based on the data extractions was developed.
3. Lastly, a refined set of interpretive themes was developed from the a priori and emergent themes for effective management of obesity in men, and the barriers and facilitators for engaging in weight management programmes. This interpretive process resulted in the development of a logic model (see figure 1) to organise the interpretive themes according to the realist and socioecological principles underpinning this research. The themes were categorised around their micro, meso and macro context, and how these influence men's perspectives and experiences related to engaging with weight management programmes.

Following the completion of the analytical cycle for studies linked to interventions, studies not linked to interventions were then read to ascertain if they provided disconfirming evidence or added any new perspectives and all relevant data were extracted. This process tested the robustness of the synthesis and has been recommended elsewhere.^23^

## Results

**Figure 2:**
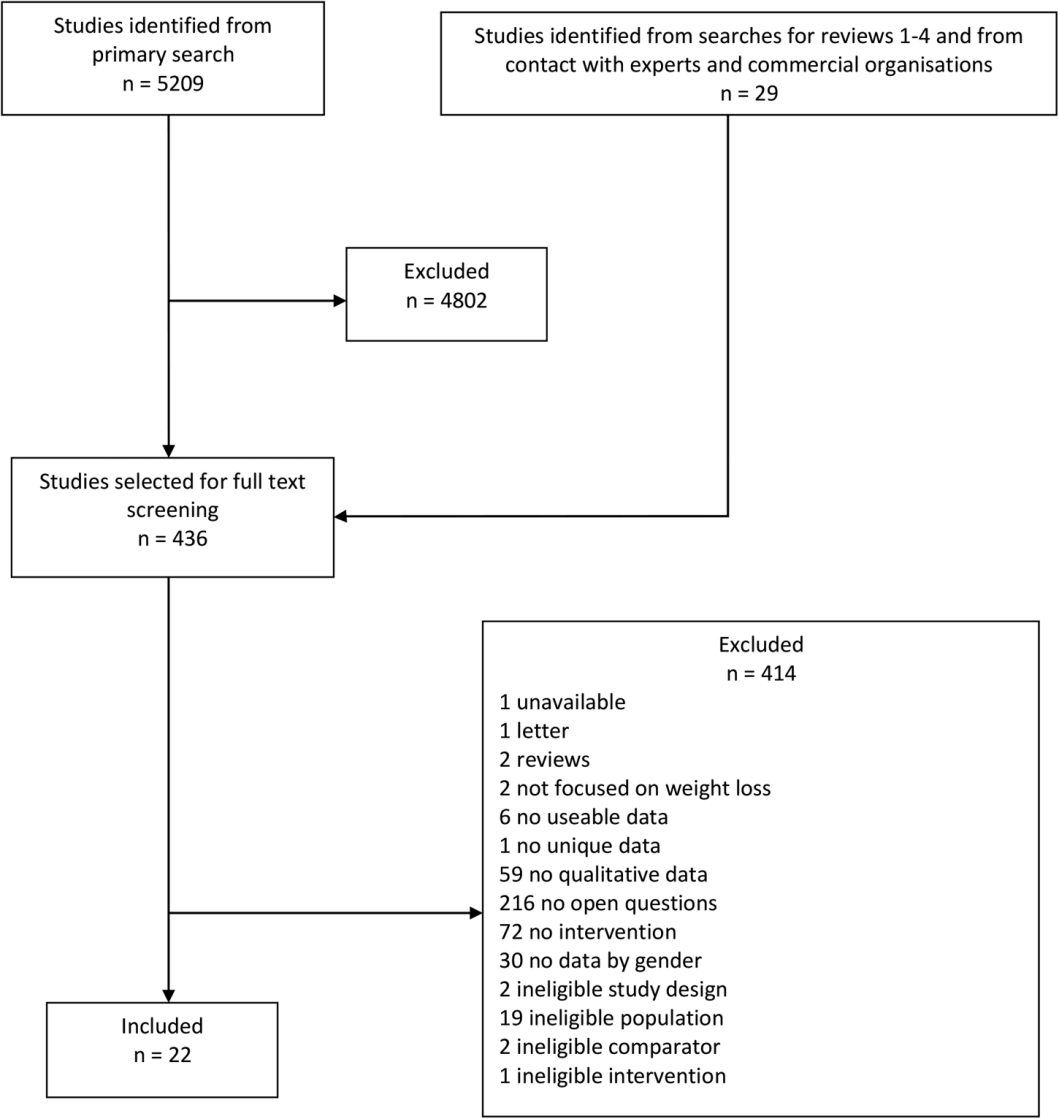
Flow chart.

Twenty-two studies were included (figure 2). Eight studies were linked to interventions that were open to men only.^9^ ^12^ ^24–29^ Of these eight, two studies^25^ ^29^ were linked to the same group-based programme, the Camelon model, which was delivered in men's health clinics in Scotland, while two other studies^12^ ^28^ were linked to the Health of Men (HoM) workplace-based weight management programme that was delivered in England in a group format. One study was linked to a group-based intervention that was delivered at Leeds Rhinos rugby league club in England.^26^ Two studies were linked to the ‘SHED-IT’ trial that was an individual website-based weight management programme delivered in Australia.^24^ ^27^ One study was linked to the group-based RCT ‘Football Fans in Training’ (FFIT) delivered in Scotland at the stadiums of senior Scottish football clubs.^9^

The remaining five studies were linked to interventions that were open to both men and women. One study^30^ was linked to a group-based RCT delivered in Australia, while a second^31^ was linked to an individual-based RCT delivered in Australia. Another study^32^ was linked to a group-based insurance-sponsored weight management scheme delivered in the USA. In addition, a further study^33^ was linked to a faith-based group weight management programme delivered within a rural African-American community. A final study^34^ analysed the experiences of individuals taking the medication orlistat for weight loss in England.

Each of the nine studies not linked to interventions was UK-based and collected primary data from a male-only sample.^7^ ^35–42^ These studies drew on the views, attitudes and perceptions of men who had prior experience of taking part in formal weight management programmes and interventions, or who have attempted to reduce or manage their weight in other ways.

A summary of key included study characteristics linked to interventions is given in table 1, while table 2 provides further details of studies not linked to interventions.

**Table 1:** Characteristics of included studies linked to interventions

Study ID and name of intervention	Intervention name	Intervention details	Linked to RCT or non-randomised intervention?	Aims	Setting (country where study was undertaken)	Group or individually delivered?	Sample size	Data collection	Data analysis
Abildso *et al*^32^	Insurance-sponsored weight management programme	Twelve-week insurance-sponsored cognitive behavioural weight management programme	Non-randomised intervention	To qualitatively explore factors associated with programme adherence and weight loss	Health service (USA)	Group	n=3 men, n=8 women	Telephone interview	Grounded theory
Gallagher *et al*^30^	HEELP	A multicomponent group-based weight loss intervention designed to follow cardiovascular disease and diabetes disease management programmes	RCT	To understand perceptions and experiences of managing weight loss in people with multiple CVD risk factors/existing CVD undertaking a WLP in Australia	Health service (Australia)	Group	n=25 men, n=10 women	Focus group	Thematic analysis
Gray *et al*^25^	Camelon model	Group-based weight management programme for obese men in a deprived area of Scotland led by community nurses and dietician	Non-randomised intervention	Extent to which Camelon model reached target population; characteristics of participants; weight loss outcomes; and participants’ views of the programme	Health service (Scotland)	Group	n=24 men	Focus group	Thematic analysis
Harrison^28^	HoM	Six-week WLP, sessions run in work place by HoM community nurses with guest sessions from dietician and activity specialist	Non-randomised intervention	Case study of one participant attending HoM programme	Workplace (England)	Group	n=1 man	In-depth interview	Thematic analysis
Hunt *et al*^9^	FFIT	A pedometer-based walking programme, part of a weight management intervention delivered through Scottish Premier League football clubs	RCT	To explore men's views of weight management intervention delivered through football clubs, and congruence or challenge this poses to masculine identities	Scottish Premier League football clubs (Scotland)	Group	n=27 men	Telephone interview	Thematic analysis
Leishman^29^	The Camelon model	A group-based weight management programme specifically for obese men in deprived area of Scotland led by community nurses and dietician	Non-randomised intervention	To explain how Camelon model functions	Health service (Scotland)	Group	Unclear	Unclear	Unclear
Mallyon *et al*^31^	VLCHF vs HCLF diet	Clinical trial that compared a LCHF and HCLF WLP	RCT	Exploration of men's experience of dieting within social context, paying attention to how differences in reflexively gendered habitus affect dieting	Health service (Australia)	Individual	n=8 men	Semistructured interview	Grounded theory
Morgan *et al*^27^	SHED-IT	An internet-based WLP exclusively for men	RCT	Perceptions and experiences of men in SHED-IT RCT. (1) what attracted them to programme, (2) satisfaction with programme/its components (3) suggestions for improvements to SHED-IT	Health service (Australia)	Individual	n=18 men	Semistructured interview	Thematic analysis
Morgan *et al*^24^	SHED-IT	An internet-based WLP exclusively for men	RCT	Process evaluation with internet group participants using quantitative (website use/questionnaire) and qualitative (interviews) data	Health service (Australia)	Individual	n=12 men	Mixed-methods: open-ended questions on questionnaire and semistructured interviews	Thematic analysis
Kim *et al*^33^	The WORD	A faith-based weight loss intervention using a community-based participatory research approach	Non-randomised intervention	Describe process behind conception of a weight management intervention, its implementation, and impact on participants	Church-based (USA)	Group	Unclear	Focus group	Grounded theory
Ogden and Sidhu^34^	Orlistat	Orlistat drug that acts on gastrointestinal system by reducing fat absorption	Non-randomised intervention	Examine patients’ experiences of taking orlistat to explore adherence and behaviour change	Health service (England)	Individual	n=4 men, n=8 women	Semistructured interview	IPA
Witty and White^26^	Tackling men’s health	A multicomponent targeted intervention on men's self-reported engagement with health services	Non-randomised intervention	Assess engagement in weight management intervention targeting men attending rugby matches	English Rugby League club (England)	Group	n=20 men	Semistructured interview	Thematic analysis
White *et al*^12^	Health of men (HoM)	A 6-week WLP sessions run in work place by HoM community nurses, with guest sessions from dietician and activity specialist	Non-randomised intervention	To explore why men would want to take part in HoM initiatives	Workplace (England)	Group	n=10 men	Semistructured interview	Thematic analysis

CVD, cardiovascular disease; FFIT, Football fans in Training; HCLF, high calorie low fat; HEELP, Healthy Eating and Exercise Lifestyle Program; HoM, Health of Men; IPA, interpretative phenomenological analysis; LCHF, low carbohydrate/high fat; RCT, randomised controlled trial; VLCHF, very low calorie high fat; WLP, weight loss programme.

**Table 2:** Characteristics of studies not linked to interventions

Study ID	Aims	Methods	Data analysis	Sample size	Country where study was undertaken
De Souza and Ciclitira^35^	Explore issues regarding men's health, with a specific focus on men's experiences of dieting	Semistructured interviews	Grounded theory	n=8 men	England
Gillon^38^	Understand implications of how men talk about weight for those working in counselling field	In-depth interviews	Discourse analysis	n=8 men	Scotland
Gough and Conner^7^	Analysis of men's accounts of food and health using concepts pertaining to masculinity	Semistructured interviews	Thematic analysis	n=24 men	England
Gough and Flanders^36^	Examine how members of gay ‘bear’ community normalise ‘excess’ weight against the backdrop of obesity ‘crisis’	Semistructured interviews	Grounded theory and thematic analysis	n=10 men	England
McCullagh^39^	Understand lifestyles of long distance lorry drivers to inform appropriate health education strategies to encourage health awareness, access services and attain a healthier lifestyle	Open-ended commentary provided on a voluntary basis following the completion of a survey	Unclear	n=168 men	England
Monaghan^40^	Contribute sociologically to burgeoning critical obesity studies that critique social construction of overweight/obesity as a public health crisis by questioning economics, science, morality and ideology of current obesity epidemic claims	In-depth interviews	Thematic analysis	n=37 men	England
Monaghan^41^	Critical realist contribution to obesity debate on men's justificatory accounts for levels of body mass that medicine labels too heavy (implicitly or explicitly too fat)	In-depth interviews	Thematic analysis	n=37 men	England
Monaghan^42^	Explore men's talk about physical activity, weight, health and slimming	In-depth interviews	Thematic analysis	n=37 men	England
Weaver *et al*^37^	Explore understandings that men in general population hold about weight, exercise and health to inform process of health promotion and diabetes prevention in men	Semistructured interviews	Thematic analysis	n=17 men	England

### Engaging in a WLP or intervention

#### Initial motivation to lose weight

Being labelled ‘obese’ and having health concerns were primary motivators for men seeking help with weight loss. Receiving a diagnosis or being labelled obese seems to act as a powerful ‘cue to action’ for many. For example, Gray *et al*^25^ found that men only experienced dissatisfaction with their body image when they were labelled obese. In contrast, men whose BMI was in the overweight range (25–29 kg/m^2^) were less likely to enrol in the study's weight management programme:

The word obese, that's what got to me. Where I work most of the guys are on the big side. My size just seemed normal. When the girl (assessing nurse) showed me the chart I was really shocked to see that I was clinically obese. If it had showed me as being fat it wouldn't have got to me as much and I probably wouldn't have done anything about it. (Age 38)

Gray *et al*^25^ therefore contended that contemporary social norms that place emphasis on the idea that men should be bigger and stronger than women have perhaps influenced men's perceptions of an ideal weight as being in the overweight range.

Anxieties and fears associated with obesity exacerbating health issues or contributing to the ageing process also seem to motivate men to lose weight.^27^ ^30^ Hospital admission or experience of what was perceived by some participants to be a life-threatening coronary or respiratory event were motivators to lose weight in Ogden and Sidhu's study^34^ of men who were taking weight loss medication.

In addition, experiencing feelings of being ‘unhealthy’ was found to be an important motivator in White *et al*’s^12^ workplace-based weight management study.

These themes associated with initial motivation to lose weight were supported in the non-intervention studies. For example, two studies^7^ ^35^ found men were likely to assert health concerns when talking about weight loss. De Souza and Ciclitira^35^ found men believed women were more motivated by concerns for their appearance than men:

I think women tend to do it a lot more for the looks. I think men as opposed to women because he has to. His legs and heart's going. Maybe for medical reasons for the man, whereas for a woman nine times out of ten she wants to get into her bikini for her holiday. (Age 54)

However, in the same study^35^ found that gay men talked about weight loss as a way to improve appearance. Slimness was described as something that was socially prized within the gay community:

I did notice I was more attractive to sort of more people on the gay scene (after losing weight) there's quite an emphasis there…yeah to have like a flat belly. (Age 33)

Nevertheless, those identifying themselves as gay ‘bears’ in the study by Gough and Flanders^36^ refuted the notion that being labelled as obese induces motivation to lose weight. In this particular gay subculture, obese bodies are viewed as desirable and healthy.

### Factors that attract men to participate

Being able to undertake weight loss activities in a male-only environment was appealing for some men. For example, in the study by Gray *et al*,^25^ a participant explained why he was attracted to the programme:

I thought I'll go along ‘cos it was all blokes anyway, ‘cos I wasn't going to go, with all due respect, with the women. (No participant characteristics provided)

A male-only environment was also found to be appealing to some participants in the study by Morgan *et al*.^27^ For example, one participant commented:

I think it was the fact that it was specifically targeted at a male only.’ (Age 21)

Nevertheless, while it appeared that some men preferred male-only features of WLPs, in the study by Morgan *et al*,^27^ there were also accounts of this being less important to other men. For example, a participant in this study^27^ mentioned that:

It didn't matter to me that it was men only, the program just sounded doable. (Age 19)

The proportions of participants who preferred a male-only to a mixed sex environment are therefore unclear from the qualitative data included in the studies aforementioned.^9^ ^12^ ^27^

Programmes that incorporated humour or ‘banter’ were found to be an important engaging factor in several studies.^9^ ^12^ ^25–29^ For example, a nurse in Witty and White's^26^ study noted that humour was important when approaching overweight or obese men about taking part in a programme as it lessened the chance of causing offence.

Morgan *et al*^27^ explained how humour was used in the ‘SHED-IT’ programme. For example, comical language and imagery, such as the picture of a beer glass, were used in the programme's promotional materials to attract men and convey the message that their internet-based programme allowed treats like beer. The theme of humour is considered again later when considering the nature of the interactions within WLPs.

A further factor that attracted men to the Camelon was the knowledge that it had been successful for other men, illustrated by this quote taken from an evaluation study:^29^

I wasn't that keen in attending a group neither but they said it worked well and is usually a good laugh so I agreed to give it a try. (No participant characteristics provided)

Engagement was also influenced by perceptions of what programmes would involve. Two studies indicated that men were disinclined to undertake any forms of strict dieting,^25^ ^29^ illustrated by the following quote from Leishman:^29^

The guy (assessing nurse) asked if I would be interested in attending a weight management programme. I agreed to attend but was a wee bit worried that it was going to be some sort of diet where you starve. (No participant characteristics provided.)

### The importance of location and setting as a ‘hook’ to engage men

The location and setting of certain programmes acted as an important element to attract men to participate in a programme. In the case of the study by Hunt *et al*^9^ the intervention was located within stadiums of Scottish Premier League (SPL) football clubs and targeted obese supporters. The aim of weight loss therefore becomes congruent with a strong personal commitment to being a football supporter. Our interpretation is that associating long-standing loyalty, commitment and pleasure attained from collectively supporting a football team (historically a male activity) with a challenging lifestyle behaviour to change like obesity, could hypothetically increase the likelihood of ‘contagious motivation’, whereby motivation for turning up to support the team either consciously or subconsciously transfers to motivation to lose weight with fellow team supporters. To support this interpretation, a participant emphasises his lifelong commitment and the incentive that this provides:

It (FFIT) was ideal for me…I wanted to actually join it because see the guys that are sort of similar to myself, put on a wee bit weight…But obviously being a [club] supporter all my life, that was a big plus as well, because even talking to people saying “oh it was like I'm going to be on this 12, 13 week course, but it's with [club]”, so that was an incentive as well. (No participant characteristics provided)

Hunt *et al*^9^ contended that the setting acted as a ‘hook’ and an additional incentive to attracting men to participate in weight loss activities which they had felt unable to do in other contexts. The football context confirmed their male identities and made them feel comfortable in this setting. Similarly, Witty and White^26^ studied a WLP in a rugby stadium, which showed that certain men seemed more inclined to attend a weight management programme located in such a setting and suggest that reduction of anxiety might be a mechanism:

it is something that I would recommend to a friend because I think it feels, um…what's the word, I think it feels more comfortable than a traditional health service, I think that even in this day and age, I think that the average member of the public still experience some significant degree of anxiety or apprehension about consulting a health professional in a building that is very clearly a health oriented building so I think that the opportunity to be able to access some form of health service in an environment that I would imagine feels far less threatening. (Age 35–44 years)

Juxtaposing a challenging task like weight loss with an activity which increases well-being might help to overcome some of the emotional barriers like anxiety and fear which could impact on enrolment and engagement with interventions delivered in a health setting.

Furthermore, White *et al*^12^ reported on men's experiences of a weight loss intervention based in the workplace, which again appeared to act as an attractor for several of the men who took part. For example, one participant states:

it was something I was looking to do whilst at work. I probably wouldn't do it out of work. Because it was inside work that was a factor for me…It's passed my mind quite a few times but I think because it's during work hours it's given me more motivation because I probably wouldn't do anything in the evenings myself. And that's helped a lot for me. (Age 54)

White *et al* stated that the convenience factor of having the programme in the workplace played a key part in attracting men; however, they also highlighted that the success of male participation in work-based programmes is to some extent dependent on the creation of a positive environment within the organisation to create the right climate for the initiative to work. In addition, one aspect that links these three separate contexts (football or rugby stadium, work environment) is that they fit well with masculine identities. Hunt *et al*^9^ noted that when a context is congruent to masculine ideals and not challenging, attempts at engaging in weight loss and health improvement activities are more palatable for men.

### Men's perspectives and experiences of weight management programmes

Once engaged and participating in weight management programmes, the content, format and delivery processes relate to the individual, biological and social determinants of health and well-being. A degree of overlap was observed in some studies regarding themes relating to engagement, since the demarcation of themes is a dynamic rather than categorical process.

#### Men and diets

Men may be inclined to avoid what is perceived as the feminised realm of dieting, where women are often viewed as experts.^36^ In addition, unpalatable diets emphasising smaller portions are also blamed for men distancing themselves from dieting.^7^ Little is known about the subjective experiences of dieting men or the meanings they attach to food, or indeed their experiences and understanding of food.^7^

Experiences of men dieting as part of a WLP were prominent in two of the included studies. For example, the approach of the Camelon weight management programme was to de-emphasise the role of dieting and weight loss, and emphasise a personalised approach that accounts for individual preferences, in order to make men feel in control of their weight loss. This approach was perceived positively by a participant in the study by Leishman:^29^

I was pleasantly surprised to find out that this was not a diet I was on but actually a course to educate us men on eating healthier, the need to be more active and to control our portions. At first I didn't think it would work for me because I felt I was eating the same amount of food I always had, just more fruit and veg. (No participant characteristics provided)

A de-emphasis on the role of extreme dieting was also employed in the SHED-IT internet-based study^27^ which appeared to be welcomed:

It's the opposite to those sorts of crash diets. Like they impose on you what you're going to eat, I was thinking oh gee, this sounds really good. (Age 35)

The SHED-IT programme also encouraged participants to enjoy some treats such as ‘beer’ and ‘junk food’ in moderation, which was perceived positively:^27^

I think the most enjoyable aspect was the fact that it allows for those days where you know, if you have a…day at work you can just go and have a few beers afterwards and not feel…house for it. (Age 21)

Thus, men from two studies^27^ ^29^ appeared to welcome aspects of programmes that placed less emphasis on strict dieting and emphasised personal control over which foods they consumed. This supports the notion that men are reluctant to diet and are attracted to participate, engage and adhere to programmes that are realistic and could feasibly be assimilated into their lives.

### Influence of partners, family and friends on men's engagement with a WLP

Hegemonic masculinity refers to a dominant form of masculinity within a gender hierarchy that focuses on practices that attempt to guarantee the dominant social position of men and the subordinate social position of women. In Mallyon *et al*'s^31^ study, men who were stated to engage in hegemonically masculine patterns received ‘appropriate’ dieting support by female partners to prepare their food, which helped men to stick to a weight management programme, while those who displayed less hegemonically masculine patterns were more likely to take control of their own dieting practices. For example, a participant, described as being more engaged with hegemonic masculinity explained his partner's role in his dieting:^31^

…my wife is a real Trojan as far as meals and that sort of thing are concerned, because she knows how to cook and she does it all for me, she doesn't have to, but she does. (Age 60–64 years)

Mallyon *et al*^31^ also provided an example of family having a negative effect on men's engagement with a WLP. Some men reported finding it difficult to refuse their mother's cooking, which might include large portions or high calorie food, fearing that any rejection would be perceived as an insult which would damage the mother–son relationship.

Mallyon *et al*^31^ found that attempts to stick to dieting plans were also vulnerable to ‘social sabotage’. For example, the attitudes of non-dieting male friends or peers were found to have an important negative or positive effect on the male dieters’ motivation to stick to a programme, which may diminish if he felt unsupported in his aim of losing weight. Unlike the facilitating influence of cohesive group activity settings like sports clubs or the workplace on the engagement of men in weight management interventions, the influence of groups such as family and friends can serve as either a facilitator or a barrier to engagement.

### Alcohol and obesity

Alcohol was considered a particular problem for men in relation to weight gain in one study,^28^ which supported a perceived causal link between alcohol consumption and increased appetite. For example, one 34-year-old man suggested that after a few beers he lowered his defences and ate more. He would have a few beers most nights and put his feet up to watch television; he commented that with the drinking his diet also deteriorated.^30^

Perceived programme flexibility was welcomed in the SHED-IT programme, which encouraged participants to enjoy some treats such as beer and junk food in moderation. One 35-year-old man used a variant of the expression having one's cake and eating it, when referring to having your beer and losing weight.^27^ In addition, a 21-year-old highlighted that:^27^

the most enjoyable aspect was the fact that it allows for those days where you know, if you have a…day at work you can just go and have a few beers afterwards and not feel…house for it. (Age 21)

This supports the notion that men are reluctant to diet and are attracted to participate, engage and adhere to programmes that seem realistic and could feasibly be assimilated into their lives. Indeed Morgan *et al*^27^ noted that most of their participants enjoyed the fact that a strict diet was not involved.

### Men and physical activity

It is argued that men are more likely than women to use physical exercise than dieting to control their weight.^9^ ^25^ However, we found very few accounts of men's views being sought on the role of physical activity. Most data were provided by one study.^9^ It is suggested by the authors that men may view physical activity in different ways to women, specifically with regard to using physical activity to become stronger, fitter and healthier,^10^ and also in how they use pedometers for self-monitoring.^9^

Pedometer use appeared to have been a key motivator for many.^9^ In particular, pedometers appeared useful in encouraging men to meet prespecified activity targets. One quote highlighted a man competing with himself to reach his daily target of recommended steps, which in turn, changed his perception of walking:^9^

I'm walking places I'd just never have dreamed of walking. (No participant characteristics provided)

The non-intervention study by Weaver *et al*^37^ provided data that confirmed men’s enjoyment of exercise. Their participants spoke of experiencing various immediate benefits, such as being more alert or being less stiff.

### Understanding interactions within a weight management programme

#### Group-based programmes and social support

Several studies highlighted the importance of group-based weight management programmes, as facilitators of peer or social support among people with similar health problems. This was observed in the study by Leishman^29^ in Camelon. Here, men praised the support they got from each other and valued the opportunity to talk to other men on a similar programme.

A 34-year-old participant in the study by Harrison^28^ was surprised at how unexpectedly supportive his work colleagues were of his participation in the HoM work-based programme. Leishman^29^ also found factors such as group competitiveness and team spirit motivated men to meet their goals.

#### Men and the use of humour

Group camaraderie to facilitate the sharing of information and humorous banter can help men to meet weight loss targets and reduce attrition.^43^ Humour and banter were valuable in building positive relationships between group members and in promoting adherence to the programme once engaged. Thus, a participant quoted by Gray *et al*^25^ explained how the camaraderie and enjoyable conversation in the group made men want to return the following week. This connects with earlier discussion where we identified the value of employing cohesive, task-oriented groups for men engaging in a process of weight management. A participant in a linked study delivered to all-men groups in a health clinic setting perceived that being with men who were in the same situation as himself made him realise he was not alone.^29^

A participant in Morgan *et al*^24^ explained that the male-only feature of the SHED-IT programme helped to facilitate engagement in male-oriented banter that, for him, would have been curtailed in a mixed-sex environment.

#### Accountability and adherence

Adherence is a decisive factor in predicting success for participants undertaking weight management programmes.^44^ Various methods were employed to help participants ‘stick to’ programmes. These include strategies that involve making men self-accountable to themselves and others for their food choices. Abildso *et al*^32^ encouraged participants to use a daily food log that was checked by staff. Having to explicitly account to staff by reviewing their personal food logs was stated to be central to successful participant weight loss. Statistical analyses revealed that food logs were more frequently completed by high weight losers than moderate losers, illustrated by this quote:

What really helped me was having somebody go over the food log every day. That was the big thing; just having staff talk about things I was eating, choices I was making, maybe making a few little suggestions—that was really very helpful. (No participant characteristics provided)

Morgan *et al*^27^ reported a similar approach to awareness and self-monitoring of food intake to promote adherence. Participants in this study were randomised to either a weight loss information-only group or to a group that received weight loss information, use of an internet weight loss website and individualised support from programme staff. Both groups received a weight loss handbook that detailed a simple energy in/energy out equation, allowing participants to keep records of their energy intake balance. This was cited by the authors as a source of satisfaction and had helped participants control their weight. Men noticing that their energy count was directly related to weight gain and weight loss had also acted as a motivating factor to continue with the WLP.^27^

#### Goal setting

A further way in which participants were encouraged to stick to the programme was by setting weight loss goals. For example, a participant in the HoM study^12^ described how setting easy weight loss goals and anticipating the satisfaction of achieving the one or two stone weight loss 1 year later helped his adherence and motivation.

### Perceptions of impacts and consequences of engagement with WLPs

#### How programmes impact on partners and family members

There were many reported reflections on how aspects of programme activities indirectly impacted on the family members of participants. For example, the Camelon programme^25^ ^29^ appeared to have a positive impact on the eating habits of partners/family of participants. A focus group with female partners of men participating in this programme^25^ indicated that many had been influenced by the men's engagement with the programme. For example, the wife of one participant stated:

It certainly had an impact on myself and now I think about what I'm eating more. (No participant characteristics provided.)

### The downside of losing weight for men

Men from a range of backgrounds were keen to avoid looking too thin. Men attending the Camelon programme felt dissatisfied with their weight only when they were labelled as obese, for example, one man was quoted as saying that if he lost too much weight he would probably look ill.^29^ This sentiment was found to be universal among the men interviewed by Gray *et al*.^25^ In support of this, other included studies also found that being in the overweight range did not especially concern men,^35^ ^38^ and in some cases represented an ideal weight as men did not want to become too thin.^33^ However, this sentiment was not universal in all of the included studies. For example, a participant in the study by White *et al*^12^ stated that he associated being overweight with being unhealthy, which was the key driver for him joining a workplace-based WLP.

### Improvements in health

Abildso *et al*^32^ and Hunt *et al*^9^ reported that many participants experienced benefits after participating in the programme, such as better quality sleep, decreased pain, improved blood pressure, improved cholesterol, loss of leg neuralgia and a decrease in headaches associated with discontinuing some of the medications taken for obesity-related conditions.

In addition, there were accounts of improvements in physical fitness as a consequence of a WLP, with positive consequences for health, for instance having a more positive mind set, feeling younger or being able to walk upstairs again.^9^

These accounts of improved perceptions of health and well-being are positively reinforcing for both adhering to the programme and to sustaining behaviour change after the programme has ended. They illustrate the sense of achievement and the motivation resulting from successful weight loss.

### Fears of relapse when programme ends

White *et al*^12^ described concern that the group may gradually stop meeting after the programme ended, which could impact on adherence to the lasting messages of the programme. In particular, a participant expressed doubts about group members continuing to walk without the group support, in the context of busy working lives. Three to six monthly group reunions were seen as a good idea to review progress.

## Discussion

This realist qualitative evidence synthesis suggests that men were motivated to lose weight once they perceived that they had a problem with their health, and were diagnosed as obese by a health professional or labelled with the term ‘obese’. Feeling unhealthy and the desire to improve personal appearance were also important motivators to lose weight in men.

Interventions in community settings, particularly associated with football or rugby clubs, or workplace settings were acceptable and attractive to men and preferable to interventions in hospital or healthcare settings. Careful use of humour in promotional materials in WLPs, such as comical language and imagery, attracted men. Furthermore, men did not want to undergo strict or extreme diets, while some men clearly wished to attend a men-only group. Nevertheless, evidence exists in the studies to suggest that engaging with a programme that is ‘do-able’ is more important to some men than whether or not the programme is open to men only and the review could not establish whether the majority of participants preferred men-only WLPs.

The review has also found that men were more likely to adhere to a WLP if the programme involved goal setting, physical activity, self-monitoring of diet, accountability and peer support.^16^ Partners were also found to have a pivotal role in successful weight loss attempts, providing support for those choosing against the expected social norms. But the influence of family members and peers who respond in a negative way can be detrimental to men's efforts to lose weight. It is important to note that a number of the above findings can also be applicable to women;^45^ ^46^ however, this review was not designed to answer questions about WLPs for women.

### Strengths and weaknesses of the study

This is the first synthesis of qualitative studies investigating men's perceptions and experiences of weight management services. Exhaustive searches were undertaken with the aim of identifying all relevant published and grey literature; however, current indexing by databases makes searching for male-related/female-related issues very challenging.^17^ As a result, it was difficult to find unpublished evaluations, and it is therefore unclear what impact this has had on the study results. In addition, the evidence base for black or ethnic minority men; men with low incomes or who are unemployed; men living in rural and/or remote areas; or men with a gay, bisexual or transgender background was limited. This review was completed in 2013; however, an updated search was conducted in June 2015 and found one relevant study that has been published since the completion of this review.^47^ However, there are no insights in this study that contradict or add anything new to the findings of this review.

### Implications for clinicians and policymakers

The findings of this qualitative evidence synthesis may help with the formation of policies to increase men's uptake and continuation with WLPs. Health issues were important intrinsic motivators, because of increased levels of concern, and extrinsic motivators, as a result of advice from others, to engage in programmes. Although health service staff can help motivate change, setting programmes in the health service may be far less attractive than using settings, such as a football stadium, that provide long-term social support and an ambience, for example, humour and banter, that appeals to men. Dieting, particularly strict dieting, is seen as a feminine activity. Thus, although reducing diets are needed for greater weight loss, strict diets seem unpopular and terms such as ‘healthier eating’ (which allows for treats such as alcohol) and ‘portion control’ seem to be more appealing to men. Although physical activity was found to be preferable to dieting in the quantitative systematic review evidence,^16^ the qualitative data was mixed.

Men may have particular difficulties perceiving that they are overweight or obese, because of the desirability of muscularity and the masculinity of a large body size. The review has found that a key motivator for men to join a WLP is a diagnosis of obesity as opposed to simply being overweight.

The need for men's health strategies in member states has been highlighted by a European Commission report^13^ which called for policy, research and practice to be developed specifically for men, whose health may be even more disadvantaged by deprivation.

The Men's Health Forum for England and Wales advocates a need to focus on male-sensitive approaches to weight loss issues and has published The HGV Man Manual^48^ designed in the format of the Haynes car maintenance manuals. Recently, the Men's Health Forum have published a practical guide for those who design, deliver or commission weight loss services for men in the Haynes style^49^ based on the ROMEO findings.

### Future research

ROMEO identified a lack of consultation with men when developing or evaluating interventions, with very little qualitative research conducted. This is surprising as men are under-represented in almost all weight loss interventions. Data from the ROMEO quantitative systematic reviews show that, once committed, men are less likely to withdraw from programmes. Therefore, a focus only on lack of engagement with programmes can underestimate the benefits of existing programmes. Men should therefore be consulted on how to optimise engagement, reach and make interventions more user-friendly.

We found that health concerns may prompt contact with health service staff and motivate men to address their obesity. Research is required to examine the most effective interventions delivered at these pivotal health service encounters when an obesity-related diagnosis is made.

There is a need to reduce the instances in which second-order author interpretations are unsupported by first-order evidence in qualitative research papers. Participant details attached to quotes should provide details of the sex of the respondent.

## Conclusion

Our research shows that there is a need to focus on male-sensitive approaches to weight management that should incorporate key aspects such as locating WLPs for men outwith the health service setting and into other settings that men find more comfortable, such as football clubs, rugby clubs and the workplace. In addition, male-sensitive approaches should also incorporate appropriate humour and banter both when attempting to approach and encourage overweight or obese men to take part in a WLP to lessen the chance of causing offence. Humour and banter can also have a valuable function in building positive relationships between group members and promoting adherence to a programme once engaged with a WLP. Men are a heterogeneous group and their perspectives and preferences within the wider context of relationships, family, work and pleasure should be sought when designing weight management services. More qualitative research is needed with men to inform all aspects of intervention design, including the setting, optimal recruitment processes and reasons for, and how processes might minimise, attrition.
